# Prevalence of chronic obstructive pulmonary disease (COPD) and its associated factors among adults in Abeshge District, Ethiopia: a cross sectional study

**DOI:** 10.1186/s12890-019-0946-z

**Published:** 2019-10-17

**Authors:** Gashaw Garedew Woldeamanuel, Alemu Basazin Mingude, Teshome Gensa Geta

**Affiliations:** 1 Department of Biomedical Sciences, School of Medicine, College of Medicine and Health Sciences, Wolkite University, P.O. Box 07, Wolkite, Ethiopia; 2 Department of Nursing, College of Medicine and Health Sciences, Wolkite University, Wolkite, Ethiopia

**Keywords:** COPD, Prevalence, Risk factors, Ethiopia

## Abstract

**Background:**

Chronic obstructive pulmonary disease (COPD) is one of the major public health problems worldwide. Despite an increasing burden of COPD in the world, it is often a neglected disease in low income countries and COPD prevalence studies are rare in Sub-Saharan Africa. The objective of this study was to determine the prevalence of COPD and its associated factors among adults in Ethiopia.

**Methods:**

A community based cross sectional study was conducted from February 5 to May 20, 2019 in Abeshge district, Southern Ethiopia. A total of 734 adults aged at least 30 years were selected using multistage cluster sampling technique and included in the study. All participants were interviewed about socio-demographic characteristics, respiratory symptoms, smoking status and clinical characteristics. Moreover, all participants underwent spirometry. We defined COPD as a post-bronchodilator FEV1/FVC of less than 70%. Data were entered into Epi-data manager 4.4 and analyzed using SPSS version 23. Descriptive statistics and binary logistic regression analysis were used and *p*-value < 0.05 was considered as significant.

**Results:**

Of the 779 adults invited to participate, 734 adults (421 men and 313 women) were participated in this study. The mean (SD) age of the participants was 39.15 (± 9.36) years, within the age range of 30–75 years. The prevalence of COPD was 17.8% (95% confidence interval [CI], 15.1–20.6). Factors significantly associated with COPD were age above 50 years (adjusted odds ratio [AOR] = 1.91, 95% CI [1.10, 3.30]), being smoker (AOR = 4.54, 95% CI [2.69, 7.66]), Exposed to biomass smoke (AOR = 2.05, 95% CI [1.06, 3.95]) and poor ventilated kitchen (AOR = 4.12, 95% CI [2.67, 6.34]).

**Conclusion:**

It is evident from this study that the prevalence of COPD in Ethiopia is high. Factors such as old age, cigarette smoking, exposure to biomass smoke and poor kitchen ventilation plays a role in the development of COPD.

## Introduction

Chronic obstructive pulmonary disease (COPD) is a chronic respiratory disorder that is usually characterized by progressive and not fully reversible airflow limitation [1, 2]. It is one of the leading causes of morbidity and mortality worldwide [3] and the burden of the disease is growing [4]. Currently, COPD is the fourth leading cause of death in the world but is expected to be the third leading cause of death by 2020 [5]. More than 90% of COPD deaths occur in low and middle-income countries [6].

COPD diagnosis requires a comprehensive approach which includes spirometry, assessment of symptoms and risk factors [7]. Patients with COPD present with a range of respiratory symptoms [8]. The most common respiratory symptoms include dyspnea, cough and/or sputum production [6]. However, the clinical presentation of COPD tends to be ignored by patients and physicians until the disease reaches to advanced stage [8]. This means that the information about COPD prevalence is likely underestimated, as the results reported are not confirmed by objective lung function tests [9, 10]. Spirometry is required to make the diagnosis of COPD [5, 11] and is being increasingly used in epidemiological studies to objectively assess airflow limitation [4].

The prevalence of COPD varies widely in different countries and it represents a large public health problem in Africa [12]. However, COPD prevalence studies are rare in Sub-Saharan Africa. A Meta analysis of five spirometry-based studies from Africa reported a mean prevalence of COPD as 13.4%, ranging from 9.4 to 22.1% [12]. Another Meta analysis of nine studies from Sub-Saharan Africa reported a prevalence ranging from 4 to 25% [13]. However, only one of the nine studies used population-based representative sampling and appropriate case definition. In 2012, a study done in rural Uganda reported a COPD prevalence of 16.2% in people older than age 30 years. Higher prevalence among people aged 30–39 years was reported in this study [14].

Cigarette smoking is considered as the most commonly encountered risk factor for COPD worldwide [15–17]. It was estimated that 73 and 40% of COPD mortality is related to smoking in developed and developing nations respectively [3]. In developing countries, indoor air pollution from biomass fuels used for cooking and heating in poorly ventilated room is an important risk factor for COPD [18]. Worldwide, more than 3 billion people are exposed to biomass smoke and women are often exposed to the burning biomass fuels during household activities [19, 20]. Other risk factors of COPD include occupational exposure to dusts and chemicals, history of pulmonary tuberculosis, chronic asthma, frequent respiratory-tract infections during childhood, outdoor air pollution, poverty and genetic susceptibility [3, 16, 18].

Despite a growing burden in low and middle income countries, COPD is often a neglected disease in these countries including Ethiopia and its epidemiology is largely unknown [21]. Several studies concerning the prevalence of COPD and its risk factors were conducted in the developed countries. However, there are limited data from Africa and not available in Ethiopia. Hence, the present study was undertaken to assess the prevalence and associated factors of COPD among adults in Abeshge district, Ethiopia.

## Methods

### Study setting and design

A community based cross sectional study was conducted in Abeshge district of Gurage Zone, Southern Ethiopia from February 5 to May 20, 2019. Gurage Zone contains 15 districts. Of these, Abeshge district, which is located at about 158 km away from Addis Ababa, was selected for this study by considering population stability in a defined area. According to the current Abeshge district administrative office report, the total population of the district is estimated to be 100,424 with 51,950 are males and 48,474 are females. The district is organized into 29 kebeles and each kebele contained an estimated 4000 populations on average.

### Study population and sampling techniques

The participants of this study were adults living in the randomly selected villages who met the inclusion criteria. The inclusion criteria for this study were adults with age ≥ 30 years, resident in the study area for ≥6 months and willingness to participate in the study. Exclusion criteria were pregnant women, those who had any mental illnesses, active tuberculosis or had any contraindication to perform spirometry such as history of eye, chest or abdominal surgery in the last 1 month, myocardial infarction, haemoptysis, current respiratory infection and history of pneumothorax, emboli or aneurysms).

The required sample size for this study was calculated using statistical formula for single population proportion by considering 95% confidence interval, 4% level of precision, design effect of 2 [22, 23], 16.2% prevalence of COPD [14] and 10% non response rate. Accordingly, the final sample size was 717.

Multistage cluster sampling technique was employed to select the study participants. Initially, 5 kebeles (Darge, Jejeba, Walga, Vido and Boketa) were selected by lottery method from the total list of 29 kebeles of Abeshge district. Then, each kebele was further divided into villages. Each village contained an estimated 60 households on average and each household consisted of an estimated 5 individuals on average. Three villages from each kebele, a total of 15 villages were selected using lottery method as the final cluster of the study populations. Finally, all adults residing in the selected villages who fulfill the eligibility criteria and available during the study period were recruited in the study. This resulted in the involvement of 779 adults, which is slightly higher than the calculated sample size.

### Data collection procedure and operational definitions

The data were collected on a face-to-face interview by trained data collectors using pretested structured questionnaire. The questionnaire was prepared based on American Thoracic Society Division of Lung Diseases questionnaire (ATS − DLD −78A) [24] and British Medical Research Council questionnaire [25] and adapted to local circumstances. The questions included socio-demographic characteristics, respiratory symptoms, smoking status, behavioral and clinical characteristics. After interview, body weight and height of the study participants were measured. Height was measured without shoes using an erect height measuring device and recorded to the nearest 0.1 cm. Weight was also measured with light clothes using a standard balance and recorded to the nearest 0.1 kg. Then, Body mass index (BMI) was calculated by using the body weight and height (weight in kilograms divided by square of height in square meters) [26].

All participants underwent spirometry using a digital portable Spirometer (Spirolab MIR, Italy) based on the American Thoracic Society and European Respiratory Society (ATS/ERS) guidelines [27]. All pulmonary function tests were done by trained technicians. Before performing each measurement, all participants were rested for at least 5 min and the test procedure was clearly explained to all subjects in their local language. Moreover, spriometers were calibrated daily. A minimum of three acceptable measurements were done and the highest values were considered the best and used for analysis. The parameters used for analysis included forced vital capacity (FVC), forced expiratory volume in 1 s (FEV1) and FEV1: FVC ratio. All participants with a pre-bronchodilator FEV1/FVC < 0.8 took inhaled salbutamol (200 μg) administered via a spacer and then, post-bronchodilator spirometry was performed after 15 min. FEV1/FVC < 0.8 was used as a cutoff to perform post-bronchodilator spirometry, to avoid FVC underestimation, which could result in normal FEV1/FVC ratio [14]. In this study, the Global Initiative for Chronic Obstructive Lung Disease (GOLD) definition of COPD was used [28]. Accordingly, COPD was defined as a post-bronchodilator FEV1/FVC < 70%. Percent-predicted post-bronchodilator FEV1 values were used to assess severity of COPD by GOLD criteria as: mild (FEV1 ≥ 80% of predicted), moderate (FEV1 = 50–79%), severe (FEV1 = 30–49%) and very severe (FEV1 < 30%).

We assessed the health status of COPD patients using COPD Assessment Test (CAT) and the modified Medical Research Council (mMRC) Dyspnoea Scale. The CAT is a validated and an eight items questionnaire [29], each with a scoring ranging from 0 to 5. Hence, the CAT has a scoring range of 0–40. Moreover, the mMRC Dyspnoea Scale was used to assess degree of functional disability attributable to dyspnoea, using five scores from 0 to 4 and a higher score indicating worse health status.

### Data quality control

To maintain the quality of data, intensive training was given for all data collectors. The data collection process was supervised continuously to check for data completeness and consistency. Prior to data collection, the questionnaires were pre-tested and necessary corrections were undertaken accordingly. The spirometers were checked regularly and calibrated prior to every measurement.

All spirometric measurements were performed at a fixed time of the day and by the same instructions to ensure its validity. Spirometry results with low quality were excluded from analysis.

### Statistical analysis

Data were entered into Epi-data manager 4.4 and then exported to SPSS version 23 for analysis. Descriptive statistics were calculated and the continuous variables were presented as mean ± standard deviation (SD) whereas the categorical variables were presented as numbers and frequencies. Odds ratio (OR) together with 95% confidence interval were used to determine the strength of association between COPD and its associated factors. Binary logistic regression analysis was used to estimate the association between COPD and its predictors. Variables that had a value of *p* ≤ 0.25 in the univariable analysis were included in the subsequent multivariable binary logistic regression analysis. A value of *p* < 0.05 was considered as statistically significant.

## Results

### Socio-demographic characteristics

Of the 779 adults invited to participate, 17 (2.2%) of them were not volunteered and 28 (3.6%) people provided unaccepted spirometry and were excluded from the study. Hence, a total of 734 adults (421 men and 313 women) were participated in this study and underwent spirometry. The mean (SD) age of the participants was 39.15 (± 9.36) years, within the age range of 30–75 years. The majority, 427(58.2%) and 337(45.9%) of the participants were farmer by occupation and illiterate, respectively. The average household size observed in the study was 4.4 persons (Table 1).

**Table 1:** Socio-demographic characteristics of the study participants in Abeshge district, Southern Ethiopia, 2019 (*n* = 734)

Characteristics	n (%)
Sex	
Male	421 (57.4)
Female	313 (42.6)
Age (in years)	
30–39	415 (56.5)
40–49	203 (27.7)
50–59	87 (11.9)
≥ 60	29 (3.9)
Marital status	
Single	93 (12.7)
Married	592 (80.7)
Divorced	20 (2.7)
Widow/ widower	29 (3.9)
Religion	
Muslim	264 (36)
Orthodox	396 (53.9)
Protestant	65 (8.9)
Catholic	9 (1.2)
Educational status	
Illiterate	337 (45.9)
Primary school	224 (30.5)
High school	98 (13.4)
Certificate and above	75 (10.2)
Occupational status	
Farmer	427 (58.2)
Employee	227 (30.9)
Housewife	61 (8.3)
Others	19 (2.6)
Family size	
≤ 5	542 (73.8)
> 5	192 (26.2)
Body mass index (kg/m^2^)	
< 18.5	114 (15.5)
18.5–24.9	516 (70.3)
≥ 25	104 (14.2)

### Behavioral and clinical characteristics

Of the 734 enrolled participants, 86 (11.7%) respondents were either former or current smokers. Of all smokers, 16 (18.6%) men and 4 (4.7%) women were former smokers, whereas all current smokers were men. About 82% (602/734) of the participants (75.3% of men and 91.1% of women) were exposed to biomass smoke. Exposure to biomass smoke was significantly higher among women as compared to men (*p* < 0.001). Women were more exposed than were men to biomass smoke both in terms of hours per day and number of years exposed (p < 0.001). In 90.3% (663/734) of the total participants, dried wood was the main fuel used for cooking and heating. Furthermore, the results revealed that 150 (20.4%) participants were presented with cough as the main respiratory symptom (Table 2).

**Table 2:** behavioral and clinical characteristics of the study participants in Abeshge district, Southern Ethiopia, 2019 (*n* = 734)

Characteristics	Totals
Smoking status	
Never smoker	648 (88.3%)
Former smoker	20 (2.7%)
Current smoker	66 (9%)
^a^Years smoked	10.3 (9.28)
^a^Cigars smoked per day	6.37 (4.21)
Biomass fuel exposure	
No	133 (18%)
Yes	602 (82%)
^a^Years exposed	15.18 (8.60)
^a^Hours exposed per day	1.92 (0.98)
^b^Fuels used	
Dried Wood	663 (90.3%)
Charcol	367 (50%)
Animal dung	94 (12.8%)
Electricity	169 (23%)
Kerosene gas	16 (2.2%)
Cooking area	
Same building	145 (19.8%)
Separate building	589 (80.2%)
Kitchen ventilation status	
Ventilated	513 (69.9%)
Not ventilated	221 (30.1%)
^b^ Respiratory symptoms	
Cough	150 (20.4%)
Phlegm	133 (18.1%)
Wheeze	57 (7.8%)
Dyspnea	118 (16.1%)
Chest pain	132 (18.0%)
^b^ Chronic diseases	
HIV/AIDS	2 (0.3%)
Heart Failure	8 (1.1%)
Asthma	22 (3.0%)
Others	24 (3.3%)
No illness	710 (96.7%)
Experience of medical visit	
No visit at all	72 (9.8%)
During symptoms	644 (87.7%)
Regularly	18 (2.5%)

^a^Data are presented as mean (SD), ^b^: multiple responses are possible

**Fig. 1:**
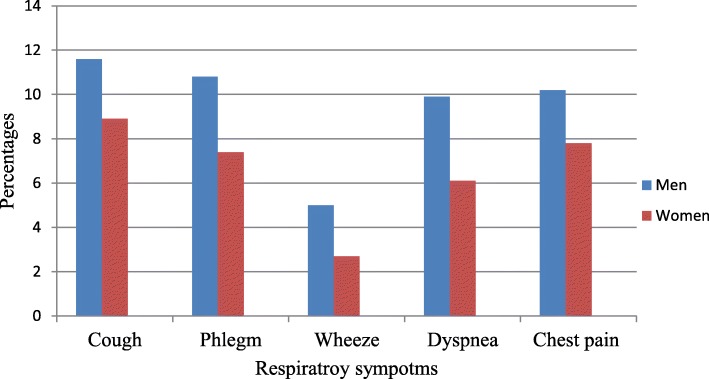
Distribution of respiratory symptoms by sex among study participants in Abeshge district, Southern Ethiopia, 2019 (*n* = 734)

We found no significant differences between men and women with regards to respiratory symptoms (all *p* > 0.05) (Fig. 1).

### COPD prevalence and risk factors

A total of 734 participants underwent pre-bronchodilator spirometry, of these; 230 participants had a pre-bronchodilator FEV1/FVC < 0.8 and underwent post-bronchodilator spirometry. Based on the post-bronchodilator assessment, the prevalence of COPD among the studied population was 17.8% (95% CI, 15.1–20.6). According to GOLD criteria, about 54.2% (71/131) of COPD patients had mild COPD. Very severe COPD was not found in this study (Table 3). Regarding to sex distribution of COPD, 70 (16.6%) of 421 men and 61 (19.5%) of 313 women had COPD. However, there was no significant (*p* = 0.317) difference in COPD prevalence across sex. Moreover, univariable analysis showed no significant differences between participants with and without COPD with regards to marital status (*p* = 0.295), co-morbidities (*p* = 0.330), family size (*p* = 0.423), educational status (*p* = 0.373), occupational status (*p* = 0.567) and medical visits (*p* = 0.31).

**Table 3:** Mean lung function parameters and prevalence of COPD according to sex among study participants in Abeshge district, Southern Ethiopia, 2019

Parameters	Sex		Totals
	Male	Female	
^a^Pre-bronchodilator spirometry (n = 734)			
FVC (liter)	3.69 ± 1.16	2.77 ± 0.98	3.29 ± 1.17
FEV1(liter)	2.91 ± 0.79	2.19 ± 0.72	2.60 ± 0.84
FEV1%	81.37 ± 14.91	81.15 ± 15.57	81.27 ± 15.18
PEF (liter/second)	4.64 ± 2.02	3.40 ± 1.63	4.11 ± 1.96
FEF_25–75%_ (liter/second)	2.92 ± 1. 34	2.22 ± 1.21	2.62 ± 1.33
^a^Post-bronchodilator spirometry (*n* = 230)			
FVC (liter)	4.33 ± 1.37	3.24 ± 1.09	3.89 ± 1.37
FEV1(liter)	2.69 ± 0.79	2.00 ± 0.50	2.37 ± 0.79
FEV1%	64.13 ± 13.29	61.16 ± 13.35	62.93 ± 13.37
PEF (liter/second)	3.08 ± 1.14	2.26 ± 1.17	2.75 ± 1.22
FEF_25–75%_ (liter/second)	2.08 ± 0.91	1.37 ± 0.80	1.79 ± 0.93
^b^ COPD (*n* = 734)			
Yes	70 (16.6%)	61 (19.5%)	131 (17.8%)
No	351 (83.4%)	252 (80.5%)	603 (82.2%)
^b^ Severity of COPD (n = 131)			
Mild	39 (55.7%)	32 (52.5%)	71 (54.2%)
Moderate	20 (28.6%)	24 (39.3%)	44 (33.6%)
Severe	11 (15.7%)	5 (8.2%)	16 (12.2%)
Very severe	0 (0)	0 (0)	0 (0)

^a^Data are presented as mean (SD), ^b^: Data are presented as n (%), *FEV1* forced expiratory volume in 1st second, *FVC* forced vital capacity, *PEF* peak expiratory flow, *FEF25–75%* forced expiratory flow at 25–75% of FVC, *COPD* chronic obstructive pulmonary disease

Variables with *p* value ≤0.25 in the univariable analysis were further entered into multivariable binary logistic regression model to control the effect of confounding variables. Age, smoking status, exposure to biomass fuel, cooking area and kitchen ventilation status were included in the multivariable analysis. After adjusting for these variables, age above 50 years [AOR = 1.91, 95% CI (1.01, 3.30), *p* = 0.021], smoking [AOR = 4.54, 95% CI (2.69, 7.66), *p* < 0.001], Exposed to biomass smoke [AOR = 2.05, 95% CI (1.06, 3.95), *p* = 0.032] and unventilated kitchen [AOR = 4.12, 95% CI (2.67, 6.34), p < 0.001] were significantly associated with COPD (Table 4).

**Table 4:** Factors associated with COPD among study participants in Abeshge district, Southern Ethiopia, 2019 (*n* = 734)

Variables	COPD		COR (95% CI)	AOR (95% CI)
	Yes (%)	No (%)		
Age (in years)				
30–39	58 (14.0)	357 (86.0)	1.00	1.00
40–49	45 (22.2)	158 (77.8)	1.75 (1.14–2.70)	1.52 (0.95–2.43)
≥ 50	28 (24.1)	88 (75.9)	1.96 (1.18–3.25)	1.91 (1.10–3.30)*
Smoking status				
Yes	36 (41.9)	50 (58.1)	4.19 (2.59–6.78)	4.54 (2.69–7.66)*
No	95 (14.7)	553 (85.3)	1.00	1.00
Exposed to Biomass fuel				
No	13 (9.8)	119 (90.2)	1.00	1.00
Yes	118 (19.6)	484 (80.4)	2.23 (1.22–4.09)	2.05 (1.06–3.95)*
Cooking area				
Same building	35 (24.1)	110 (75.9)	1.63 (1.05–2.53)	0.93 (0.57–1.53)
Separate building	96 (16.3)	493 (83.7)	1.00	1.00
Kitchen ventilation status				
Ventilated	56 (10.9)	457 (89.1)	1.00	1.00
Not ventilated	75 (33.9)	146 (66.1)	4.19 (2.83–6.21)	4.12 (2.67–6.34)*

*= Statistically significant (*p* < 0.05), *COR* Crude odds ratio, *AOR* adjusted odds ratio, *CI* confidence interval, 1.00 = reference group

This study showed a significant correlation between age and years of exposure to biomass smoke (r = 0.504, *p* < 0.001). Moreover, participants with COPD had more years of exposure to biomass smoke than those without COPD (16.97 ± 8.81 years versus 14.74 ± 8.50 years, *p* = 0.012). Similarly, the mean (SD) hours per day exposed to biomass smoke was significantly (*p* < 0.001) higher in patients with COPD (2.25 ± 1.13 h) as compared to participants without COPD (1.84 ± 0.92 h).

### COPD clinical presentation

**Fig. 2:**
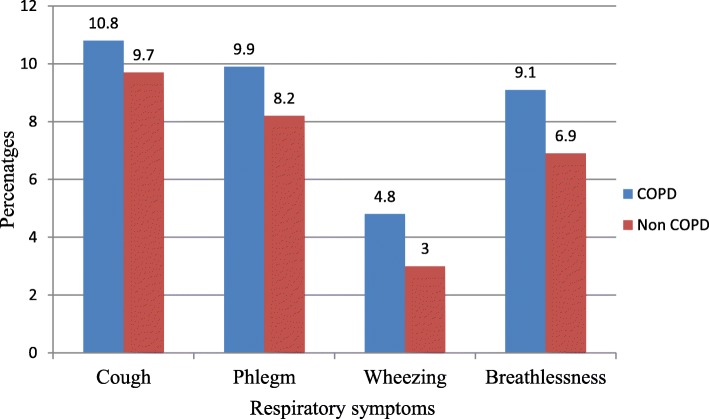
Prevalence of respiratory symptoms in patients with and without chronic obstructive pulmonary disease (COPD) in Abeshge district, Southern Ethiopia, 2019 (*n* = 734)

About 60.3% (79/131) of the participants with COPD presented with cough as the main respiratory symptom. About 55.5% (39/70) of men COPD patients and 65.6% (40/61) of women COPD patients had cough. Respiratory symptoms of cough, phlegm, wheeze and shortness of breath were significantly more common in patients with COPD than those who did not have COPD (all *p* < 0.001) (Fig. 2).

The Mean (SD) CAT score of COPD patients was 11.90 (± 3.05) with 11.80 (± 3.54) in men and 12.20 (± 2.39) in women. According to the mMRC dyspnoea scale, nearly half of the participants with COPD had no dyspnoea (score 0) (Table 5).

**Table 5:** CAT and mMRC Dyspnoea Scale in participants with COPD in Abeshge district, Southern Ethiopia, 2019 (*n* = 131)

	Male (*n* = 70)	Female (*n* = 61)	Total (n = 131)
^a^CAT total score	11.80 (3.54)	12.20 (2.39)	11.90 (3.05)
mMRC Dyspnoea Scale			
0	31 (44.3%)	33 (54.1%)	64 (48.9%)
1	22 (31.4%)	11 (18.0%)	33 (25.2%)
2	15 (21.4%)	14 (23.0%)	29 (22.1%)
3	2 (2.9)	3 (4.9%)	5 (3.8%)
4	0 (0)	0 (0)	0 (0)

^a^Data are presented as mean (SD), *CAT* COPD assessment test, *mMRC* modified Medical Research Council

## Discussion

The burden of COPD is raising worldwide [30] and it is one of the most common causes of mortality in most countries [18]. Many patients with COPD are still under-diagnosed, inadequately evaluated and under-recognized which leads to significant underreporting of the disease [18, 31]. Community based studies using appropriate methods are needed to determine the epidemiology of COPD and to inform the development of prevention and management strategies for the future. Hence, this study was aimed to assess the prevalence of COPD and its associated factors among adults in Abeshge district, Ethiopia. To the best of our knowledge, this is the first spirometry based study that reported the prevalence and risk factors of COPD in rural areas of Ethiopia.

In the present study, the prevalence of spirometry-defined COPD was 17.8% (16.6% in men and 19.5% in women). This finding is comparable with previous studies conducted in Uganda [14] and Tanzania [8], which reported prevalence of COPD as 16.2 and 17.5%, respectively. A study by Finney et al. [13] reported that the prevalence of COPD in sub-Saharan Africa varied from 4 to 25% depending on the criterion used for diagnosis. Another study by Adeloye et al. [12] reported that the prevalence of COPD in Africa ranged from 9.4 to 22.1% based on spirometry data. The PLATINO study conducted in five Latin American cites showed that the prevalence of COPD ranged from 7.8% in Mexico City to 19.7% in Montevideo [32]. The prevalence of COPD among adults was also reported in other studies from different countries. In comparison to our result, some studies reported lower prevalence [6.0% in Peru [33], 6.8% in Canada [9], 6.6% in Egypt [31], 13.4% in Korea [34] and 14.2% in Portugal [35]] and some other studies reported higher prevalence (24% in Netherlands [36], 18.4% in Greece [37] and 21.8% in Russia [38]]. In fact, the prevalence of COPD is variable across countries and across different groups within countries [18]. The differences in the current findings from other studies could be due to variations in study population, sample size, diagnostic criteria, methodology and differences in healthcare systems.

This study showed that old age (50 years and above) was significantly associated with high prevalence of COPD. This finding is consistent with the result of other studies, which reported that the frequency of COPD had a tendency to increase with age and old age is considered as a risk factor for developing COPD [3, 39–41]. The association of COPD with old age may be attributed to more exposure to risk factors and physiological decrease in respiratory function with age which begins around the age of 30–40 years [1, 41]. As a result of increase in life expectancy of the world population, a huge number of individuals will be at risk of developing COPD in the future. The changing age structure of the world’s population will contribute for the growing prevalence of COPD in recent years [3, 18].

The present study found that participants who were exposed to biomass smoke had greater odds of having COPD than non exposed participants. In line with this finding, other studies showed that people with exposure to biomass smoke are at high risk of developing COPD [39, 42, 43]. Biomass smoke is a significant contributor of indoor air pollution that leads to COPD worldwide [44]. More than 90% of the rural households depend on biomass fuel for cooking and heating in sub-Saharan Africa [20]. In agreement with our result, other studies showed that women were more exposed to biomass smoke and hence more likely to develop the disease [45, 46]. In general, participants who were exposed to biomass smoke for the longest duration and spend more hours in close proximity to biomass smoke are at risk of developing COPD [19]. The constituents of biomass fuel smoke have irritant effects on the airways [19]. It causes airway thickening due to inflammation, oxidative lung damage and protease/antiprotease imbalance which leads to the development of COPD [45]. The present study also revealed that cooking in the kitchen with poor ventilation was associated with an increased risk of developing COPD. Similar findings were reported in other studies [18, 47]. Burning biomass fuels with poor kitchen ventilation resulting in high level of indoor air pollution and exposure to a range of pollutants [48].

In this study, the prevalence of COPD in the smoker group (both former and current smoker) was found to be higher than that in the non smoker group. Similar findings were reported in several studies [1, 3, 7, 9, 41]. Cigarette smoking is the most common risk factor for COPD worldwide [3]. Other studies [14, 49] are consistent with our finding and have found that smoking history was greater in men than women. However, in the present study, the prevalence of COPD in non smokers was 14.7%, which was much higher in comparison to other studies [3.9% in Tunisia [49] and 2.83% in Egypt [31]] suggested that factors other than cigarette smoking might also be involved in COPD.

Our study has several strengths. To our knowledge, this is the first spirometry based study conducted in rural areas of Ethiopia to determine COPD prevalence using standardized pulmonary function tests. Moreover, it is a community based study with a relatively large sample size, providing a standardized measure of prevalence in Ethiopia. While many other studies included adults over 40 years of age, this study included adults with age 30 years and above to avoid missing people with air flow obstruction.

This study also has some limitations. First, we have used the fixed FEV1: FVC ratio to define COPD which could lead to over-diagnosis in older individuals and under-diagnosis in young adults [50]. Second, post-bronchodilator spirometry was not performed for all study participants. However, in order to avoid missing participants with COPD, we have used pre-bronchodilator FEV1/FVC < 0.8 as a cutoff to perform post-bronchodilator spirometry. Third, the study was based on self reported age which may contain errors. Fourth, our results indicated the situation in some rural areas and not represent the whole country. Finally, this study was cross sectional that couldn’t determine causality of possible risk factors.

## Conclusion

We found that the prevalence of COPD among adults in Ethiopia was high. In this study, the risk factors that influence the development of COPD have been identified and these include age, exposure to biomass smoke, cigarette smoking and poor kitchen ventilation. Further large scale studies with more diverse sample are needed to elucidate the impact of these risk factors and other factors on the development of COPD. Furthermore, a comprehensive national COPD disease management and prevention strategies are needed to prevent its adverse effects. The strategies could include; use of alternative clean fuels, improve kitchen ventilation and cessation of cigarette smoking.

## Data Availability

The datasets used and/or analyzed during the current study are available from the corresponding author on reasonable request.

## Abbreviations

CAT: COPD Assessment test
COPD: Chronic obstructive pulmonary disease
FEV1: Forced expiratory volume in 1 s
FEV1/FVC: Ratio of forced expiratory volume in 1 s to forced vital capacity
FVC: Forced vital capacity
GOLD: Global initiative for chronic obstructive lung disease
mMRC: modified Medical Research Council
SD: Standard deviation
